# A Novel Method for Deposition of Multi-Walled Carbon Nanotubes onto Poly(p-Phenylene Terephthalamide) Fibers to Enhance Interfacial Adhesion with Rubber Matrix

**DOI:** 10.3390/polym11020374

**Published:** 2019-02-20

**Authors:** Xuan Yang, Qunzhang Tu, Xinmin Shen, Pengxiao Zhu, Yi Li, Shuai Zhang

**Affiliations:** 1 College of Field Engineering, Army Engineering University of PLA, Nanjing 210007, China; yangdaxuan93@163.com (X.Y.); zhangshuai1236@gmail.com (S.Z.); 2 State Key Laboratory of Intelligent Manufacturing of Advanced Construction Machinery, Xuzhou Construction Machinery Group, Xuzhou 221004, China; eddyzhupx@163.com (P.Z.); cxly1965@163.com (Y.L.)

**Keywords:** poly(p-phenylene terephthalamide) fibers, multi-walled carbon nanotubes, surface properties, adhesive properties

## Abstract

In order to enhance the interfacial adhesion of poly(p-phenylene terephthalamide) (PPTA) fibers to the rubber composites, a novel method to deposit multi-walled carbon nanotubes (MWCNTs) onto the surface of PPTA fibers has been proposed in this study. This chemical modification was performed through the introduction of epoxy groups by Friedel–Crafts alkylation on the PPTA fibers, the carboxylation of MWCNTs, and the ring-opening reaction between the epoxy groups and the carboxyl groups. The morphologies, chemical structures, and compositions of the surface of PPTA fibers were characterized by scanning electron microscope, Fourier transform infrared spectroscopy, and X-ray photoelectron spectroscopy. The results showed that MWCNTs were uniformly deposited onto the surface of PPTA fibers with the covalent bonds. The measurement of contact angles of the fibers with polar solvent and non-polar solvent indicated that the surface energy of deposited fibers significantly increased by 41.9% compared with the untreated fibers. An electronic tensile tester of single-filament and a universal testing machine were utilized to measure the strength change of the fibers after modification and the interfacial adhesion between the fibers and the rubber matrix, respectively. The results showed that the tensile strength had not been obviously reduced, and the pull-out force and peeling strength of the fibers to the rubber increased by 46.3% and 56.5%, respectively.

## 1. Introduction

As a kind of artificial organic fiber, poly(p-phenylene terephthalamide) (PPTA) fiber has good integrated performances including high specific strength and elastic modulus as well as great heat resistance, corrosion properties, and toughness [1,2,3]. Therefore, PPTA fibers are often used as new types of framework material in rubber products such as hoses, synchronous belts, conveyer belts, tires, and rubber tracks [4,5], which aim to achieve the desired effect of reinforcement and weight reduction.

The molecular chain of PPTA, which is connected by benzene rings and amide groups situated in the para site, has good regularity and high crystallinity, resulting in extraordinarily smooth surface and high chemical inertness of PPTA fiber [6,7]. Therefore, a poor interfacial adhesion between fiber and rubber matrix is perceived and the integral performance of PPTA fibers/polymer matrix composites may be affected adversely. Thus, the surface modification of PPTA fibers has become a hot issue. The modification methods can be classified as physical and chemical. Ultrasonic [8], high-energy radiation [9,10,11], plasma treatment [12,13], and other physical modification methods are utilized in order to improve the physical states and enhance the roughness and wettability of the surface of fibers. Chemical modification methods including acid etching [14,15], biological enzyme [16,17], and surface grafting [18,19] can introduce active groups onto the surface of fibers or destroy the crystal states. Namely, these methods can enhance the interaction between fiber and matrix by increasing surface activity or forming chemical bonds.

Multi-walled carbon nanotubes (MWCNTs) have extremely high tensile strength of 11~63 GPa and a Young’s modulus of 270~950 GPa [20]. Moreover, abundant polar groups in the open ends of MWCNTs can expand the application range of composite materials [21]. The deposition of MWCNTs onto fibers, which is a kind of surface grafting method, has attracted extensive attention due to its ability to introduce various functional groups onto the surface of fibers to improve the mechanical property, electrical conductivity and heat conductivity [22]. O’Connor and co-workers [23] found that carbon nanotubes and aramid fibers could be efficiently dispersed and swelled in N-methylpyrrolidone (NMP), respectively. Furthermore, the experimental results indicated that the mass uptake was 4 wt.% and the mechanical property could be improved significantly. In Chen and co-workers’ research [24], the acyl chloride-functionalized MWCNTs were chemically deposited onto aramid fibers to introduce the amino groups, which also increased the single-filament tensile strength and interlaminar shear strength by 12% and 30%, respectively. Similarly, Gregory et al. [25] and Rodriguez-Uicab et al. [26] broke the molecular chain of aramid fibers to produce amino groups by hydrolysis reaction, which followed by grafting the port-functionalized MWCNTs. He and co-workers [27] had successfully grafted the aminated MWCNTs onto the carbon fiber functionalized by acyl chloride. Ku-Herrera et al. [28,29] grafted the oxidized MWCNTs onto the glass fiber by ring-opening reaction of epoxy groups in order to improve various properties of the composites.

However, few studies focused on the deposition of MWCNTs onto the PPTA fibers in the rubber matrix composites.Therefore, we tried to deposit massive MWCNTs onto the PPTA fibers to enhance the interfacial adhesion with the rubber matrix through a novel covalent deposition method.

In this research, we improved Friedel–Crafts alkylation [30] to generate the epoxy groups mildly and effectively on the surface of PPTA fibers. Meanwhile, MWCNTs were acidized to generate the carboxyl groups. With the effects of ultrasonic and basic catalyst, the carboxylated MWCNTs suspended in NMP could form covalent bonds with PPTA (namely, the epoxy ring-opening reaction) to achieve chemical deposition. The morphologies of original and modified PPTA fibers were observed by scanning electron microscope (SEM). The chemical compositions and structures of the fibers were characterized by Fourier transform infrared spectroscopy (FTIR) and X-ray photoelectron spectroscopy (XPS). The tensile strength and contact angles of the fibers were tested by electronic tensile tester of single-filament and optical microscope (OM), respectively. Furthermore, the interfacial adhesive performance of PPTA fibers with the rubber matrix was characterized by pull-out test and peeling strength test performed by universal testing machine.

## 2. Experimental

### 2.1. Materials

The PPTA fibers used in this study, which were purchased from Changzhou Gaoyuan Group Co., Ltd., Changzhou, China, were Kevlar® K-29 (dtex 3300). MWCNTs with the mean diameter of 8~15 nm and typical length of 0.5~2 μm were purchased from Nanjing XFNANO Materials Tech Co., Ltd., Nanjing, China. Epoxy chloropropane, 1,2-dichloroethane, aluminum chloride, ethanol, NMP, sodium hydroxide, potassium hydroxide, and hexane were purchased from Aladdin Industrial Co., Ltd., Shanghai, China. Sulfuric acid, nitric acid, and acetone were purchased from Tianjin Kermel Chemical Reagent Co., Ltd., Tianjin, China. All these chemicals were of analytical reagent grade and used without further purification. Table 1 shows the rubber formulation used in interfacial adhesion tests. All these ingredients were purchased from Nanjing Xinyue Chemical Industrial Co., Ltd., Nanjing, China and of industrial grade.

### 2.2. Functionalization of MWCNTs

400 mg MWCNTs were added into 200 mL mixture acid solution (H_2_SO_4_:HNO_3_ = 3:1). The mixture was first stirred at 60 °C for 15 min and then treated with ultrasonic bath of 100 W and 42 kHz for 3 h in order to oxidize MWCNTs well. After that, the treated MWCNTs were filtered by vacuum filtration equipment (Jinteng experimental equipment Co., Ltd., Tianjin, China), washed 10 times with the distilled water and dried at 150 °C for 4 h. Untreated MWCNTs and treated MWCNTs were labeled as A-MWCNTs (as-received MWCNTs) and COOH-MWCNTs, respectively.

### 2.3. Surface Modification of PPTA Fibers by Friedel–Crafts Alkylation

PPTA fibers were cleaned with 1,2-dichloroethane and acetone by turns and dried in vacuum oven at 100 °C for 3 h. 5 g aluminum chloride was added into 200 mL ethanol and then stirred for 10 min to prepare aluminum chloride-ethanol solution. Then, the dry and clean PPTA fibers (about 100 mg), 300 mL epoxy chloropropane and 200 mL aluminum chloride-ethanol solution were placed in a three-neck flask, followed by heating to 70 °C for 5 h. After that, the fibers were washed with 1,2-dichloroethane and acetone 10 times again to ensure there was no catalyst remained. The clean fibers were added into 200 mL sodium hydroxide solution (50 wt.%) at 80 °C for 2 h. Finally, the fibers after reaction were washed with the distilled water until the pH value was 7, followed by heating to 100 °C for 3 h in the vacuum oven. Untreated fibers and treated fibers were labeled as A-PPTA (as-received PPTA) and F-PPTA, respectively.

### 2.4. Deposition of MWCNTs onto Fibers

COOH-MWCNTs were dispersed in the NMP (the concentration of COOH-MWCNTs was approximately 1 mg/mL) by ultrasonic at 25 °C for 1 h in order to prepare the suspension. F-PPTA fibers were added into 300 mL COOH-MWCNTs suspension, followed by reaction for 2 h under the ultrasonic environment. Afterwards, 20 mL potassium hydroxide solution (25 wt.%) was added into the reaction liquid and then the mixture was heated to 100 °C for 2 h in the oil bath. The reacted fibers were washed 10 times with the distilled water and dried in vacuum at 25 °C for 24 h. These treated fibers were labeled as MWCNTs-PPTA.

### 2.5. Preparation of PPTA Fibers/Rubber Composites

In order to test the interfacial adhesion strength between the PPTA fibers and the rubber matrix, the fibers/rubber composites were prepared for the tests of pull-out force and peeling strength.

**Figure 1:**
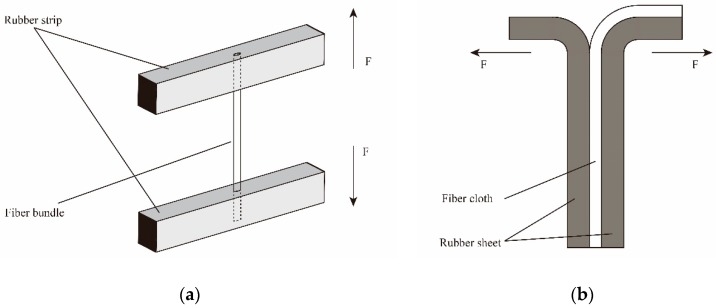
Test schematics of adhesive performance of poly(p-phenylene terephthalamide) (PPTA) fibers/rubber matrix: (**a**) pull-out force; (**b**) peeling strength.

Firstly, the rubber ingredients as shown in Table 1 were mixed in an internal mixer in order at 80 °C for 10 min. Then, the mixture was cut into some small strips and sheets, followed by placing into the testing molds of pull-out force and peeling strength, respectively. After that, PPTA fiber bundles or cloths were embedded into the molds and covered with another strip or sheet. Finally, the fibers/rubber composites were vulcanized at 140 °C for 45 min under 20 MPa. Figure 1a,b show the test schematics of pull-out force and peeling strength, respectively. Where, “F” represents the direction of the mechine force.

### 2.6. Characterization

The surface morphology of PPTA fibers was observed by SEM (Hitachi SU3500, Hitachi, Japan). FTIR (PerkinElmer Spectrum Two, Waltham, MA, USA) was used to characterize the changes of chemical structures on the surface of PPTA fibers, and the wavenumber range was 4000~650 cm^−1^. The changes of chemical compositions on the surface of fibers were characterized by XPS (ESCALAB 250, Thermo Electron Corporation, Waltham, MA, USA). The C 1s line at 284.8 eV was taken as the reference of all binding energies to compensate for the surface charging effects and Thermo Avantage v5.976 was used to fit peaks of high-resolution spectra. The contact angles were measured by drop length-height method. Firstly, a single-fiber, whose length was about 40 mm, was stuck on the object stage of OM (Nikon ECLIPSE MA100, Tokyo, Japan). Afterwards, the droplets of testing solvents (water and hexane) were deposited onto the fiber from a spray bottle. When stable droplets were formed on the surface of fiber, the images were photographed by the charge-coupled device, and finally the contact angles were fitted and measured by Young-Laplace software. The changes of tensile strength of PPTA fibers before and after deposition were tested by electronic tensile tester of single-filament (YM-06B, Shaoxing, China). The gauge length and tensile speed were 20 mm and 5 mm/min, respectively. During the testing, at least 20 specimens with a diameter of 14 μm were prepared in order to ensure the reliability of the results. The interfacial adhesive performance including pull-out force and peeling strength were tested by universal testing machine (ETM104C, Wance Testing Machine, Shenzhen, China). The tensile speed was 100 mm/min. Each test was repeated at least 8 times and the average was considered as the final result. Additionally, these specimens had the same embedded depth for fiber bundles or the same embedded width for fiber cloths to ensure the accuracy of the results.

## 3. Results and Discussion

### 3.1. Reaction Mechanism

**Figure 2:**
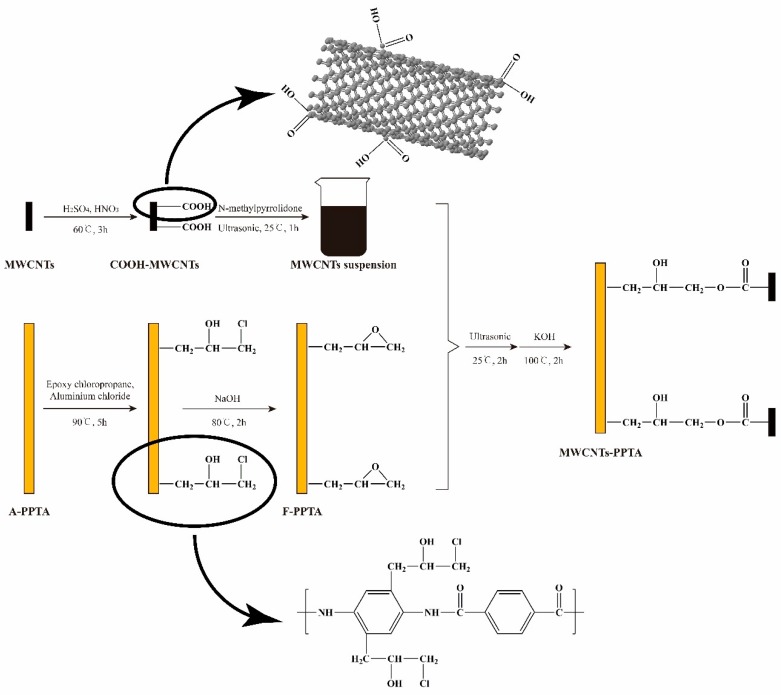
Reaction mechanism for the preparation of multi-walled carbon nanotubes (MWCNTs)-PPTA.

Figure 2 shows the deposition process of MWCNTs onto the PPTA fibers. During this deposition process, the reason for preparation of MWCNTs suspension was that MWCNTs could be efficiently dispersed in the NMP [31], which would provide an excellent reaction zone. In Friedel–Crafts alkylation of PPTA fibers, the epoxy groups could be grafted at multipoints on the benzene rings by electrophilic substitution. Furthermore, in order to effectively reduce the reaction intensity of aluminum chloride and make it fully contact with the reactants, aluminum chloride-ethanol solution was prepared to participate in this catalytic reaction. Due to the swelling of PPTA fibers in the NMP [23], the steps that F-PPTA fibers were first placed into the MWCNTs suspension followed by treating under ultrasonic at room temperature for 2 h were for the purpose of increasing the reaction contact area. After that, the addition of potassium hydroxide could catalyze the ring-opening reaction between the epoxy groups of F-PPTA and the carboxyl groups of COOH-MWCNTs [32,33]. Namely, this reaction mechanism would achieve the covalent bonding between the MWCNTs and the PPTA fibers.

### 3.2. Surface Morphologies of the PPTA Fibers

**Figure 3:**
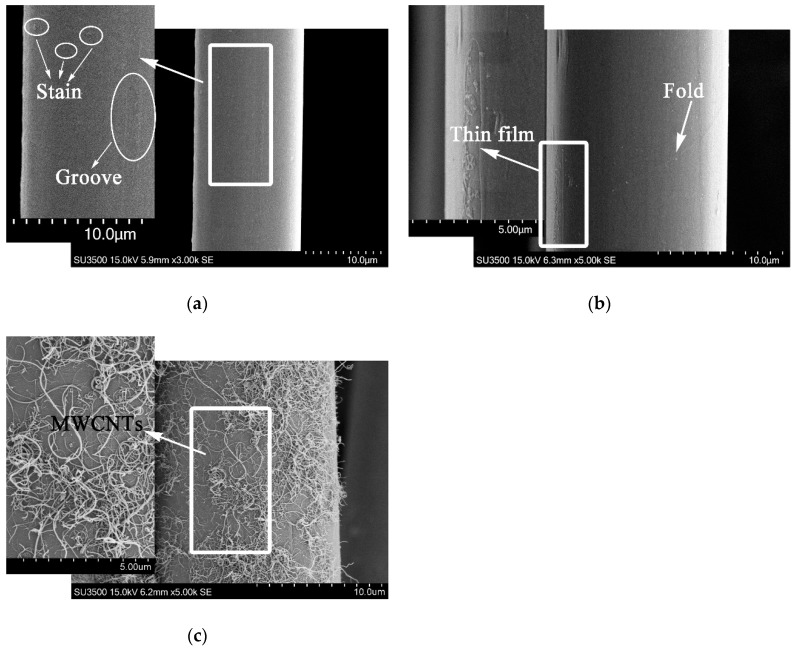
SEM images of PPTA fibers: (**a**) A-PPTA; (**b**) F-PPTA; (**c**) MWCNTs-PPTA.

It could be obtained from the weights of PPTA fibers before and after deposition that the latter was 5.6% heavier than that of the former. Namely, the effective deposition amount of MWCNTs was approximately 5.6 wt.%. The surface morphology of PPTA fibers could be observed clearly through SEM, which could provide the basis for chemical structure analysis. It could be observed from the morphology of A-PPTA as shown in Figure 3a that the surface was smooth and clean. The slight groove and stain, which were attributed to the moulage and residual size generated during drawing the production of fibers, could also be observed. Compared with Figure 3a, a new thin film was formed on the F-PPTA surface as shown in Figure 3b. The fracture of the thin film and the foldcould be observed on the left and right side in Figure 3b respectively, which might illustrate that the epoxy groups were grafted onto the surface of PPTA fibers to form such a film after Friedel–Crafts alkylation. Figure 3c shows that there were a large number of MWCNTs depositing on the surface of PPTA fibers uniformly without agglomeration phenomenon, which clearly indicated that MWCNTs were successfully grafted by this novel method.

### 3.3. Chemical Structures and Compositions of MWCNTs and PPTA Fibers

The chemical structures of MWCNTs and PPTA fibers before and after modification could be analyzed by various peaks of chemical groups through FTIR, respectively.

**Figure 4:**
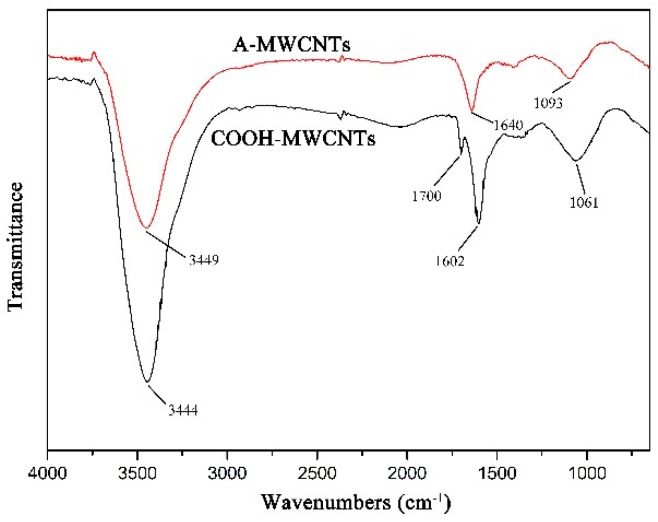
FTIR spectra of MWCNTs.

The FTIR spectra of MWCNTs are presented in Figure 4. In A-MWCNTs spectrum, the peaks at 3449 cm^−1^ and 1093 cm^−1^ were attributed to the stretching vibration of hydroxyl (–OH) and C–O on the MWCNTs surface, respectively, which might be mainly because of oxidation during the raw material purification or atmospheric moisture [34]. The peak at 1640 cm^−1^ resulted from the stretching vibration of C=O in the quinone groups on the MWCNTs surface [35]. In COOH-MWCNTs spectrum, there was a new peak at 1700 cm^−1^ attributed to the stretching vibration of C=O in the carboxyl groups (–COOH), which compared with the peaks of A-MWCNTs. Moreover, the relative intensities of the bands at 3444 cm^−1^ (–OH) and 1061 cm^−1^ (C–O) became stronger obviously. It could be confirmed from the above changes in the FTIR spectra that the carboxyl groups were generated on the MWCNTs surface after mixture acid treatment.

**Figure 5:**
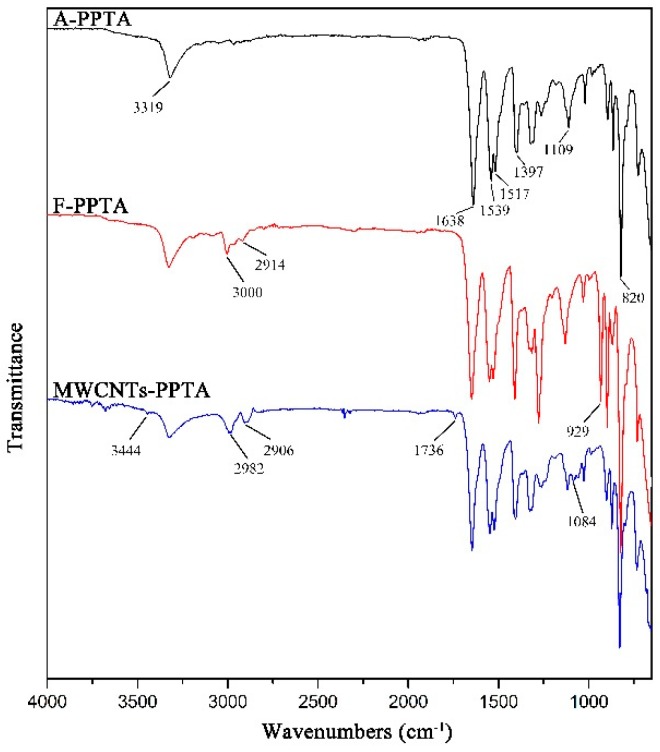
FTIR spectra of PPTA.

The FTIR spectra of PPTA fibers are shown in Figure 5. In A-PPTA spectrum, the peaks at 3319 cm^−1^ and 1539 cm^−1^ were attributed to the stretching vibration and the bending vibration of N-H bonds, respectively. The strong peaks observed at 1638 cm^−1^ and 820 cm^−1^ resulted from the stretching vibration of C=O in the amide carbonyl groups and the bending vibration of C–H, respectively. The stretching vibration of C-N was observed at 1397 cm^−1^ and 1109 cm^−1^, and the peak at 1517 cm^−1^ was attributed to the stretching vibration of C=C [36,37]. Compared with the spectrum of A-PPTA, there were new peaks in 2914~3000 cm^−1^ and at 929 cm^−1^ in that of F-PPTA, which were attributed to the stretching vibration of methylene and the stretching vibration of the epoxy groups, respectively. This could prove that the epoxy chloropropane had been successfully grafted on the PPTA fibers by Friedel–Crafts alkylation, which would provide possibilities for subsequent reactions. In MWCNTs-PPTA spectrum, two new peaks appeared at 1736 cm^−1^ and 1084 cm^−1^ were attributed to the stretching vibration of C=O and C-O-C in the ester groups, respectively. Furthermore, there was a new peak at 3444 cm^−1^ resulted from the stretching vibration of –OH. In addition, the peak ascribed to the epoxy groups disappeared. Therefore, it could be obtained that COOH-MWCNTs were deposited onto the PPTA fibers by covalent bonds.

**Figure 6:**
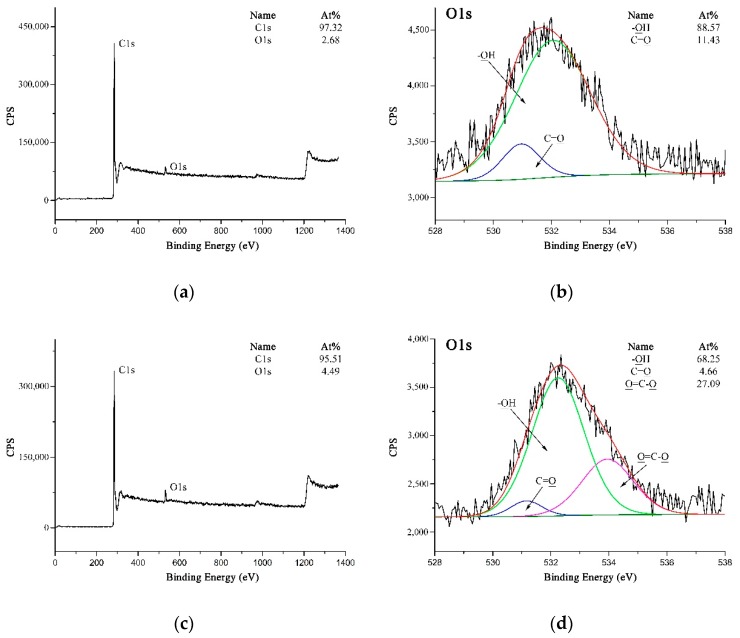
XPS spectra of MWCNTs: (**a**) Wide-scan spectrum of A-MWCNTs; (**b**) High-resolution O 1s spectrum of A-MWCNTs; (**c**) Wide-scan spectrum of COOH-MWCNTs; (**d**) High-resolution O 1s spectrum of COOH-MWCNTs.

XPS could be utilized as a suitable tool to evaluate the modifications carried out on the MWCNTs and PPTA fibers by testing the chemical compositions and structures. Figure 6 shows the XPS spectra of MWCNTs. It could be obtained from Figure 6a,c that the O 1s peak was strengthened obviously after acidification, and the oxygen concentration on MWCNTs surface increased from 2.68 at.% to 4.49 at.%. On A-MWCNTs surface, the small amount of oxygen could be attributed to the absorbed oxygen species [35]. The high-resolution O 1s spectrum of A-MWCNTs could be fitted as shown in Figure 6b with two peaks, which had binding energies at 532.05 eV for –OH (88.57 at.%) and at 530.95 eV for C=O (11.43 at.%). After acidification, there was a new peak at 533.76 eV attributed to O=C–O (27.09 at.%) as shown in Figure 6d. This could further validate that the carboxyl groups were generated on the MWCNTs surface.

**Figure 7:**
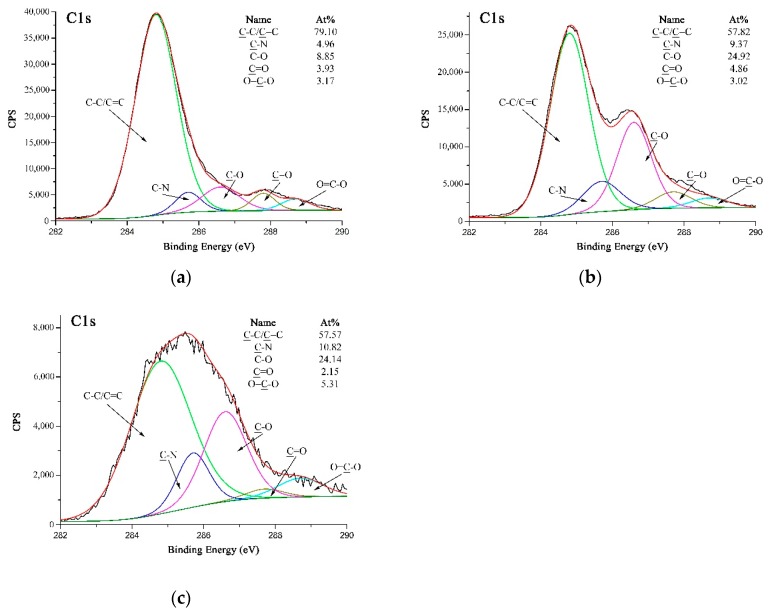
High-resolution C 1s XPS spectra of PPTA: (**a**) A-PPTA; (**b**) F-PPTA; (**c**) MWCNTs-PPTA.

Figure 7a–c are high-resolution C 1s XPS spectra of A-PPTA, F-PPTA and MWCNTs-PPTA, respectively. Figure 7a could be fitted with five peaks, which were attributed to C-C/C=C at 284.8 eV, C-N at 285.7 eV, C–O at 286.6 eV, C=O at 287.7 eV and O=C–O at 288.7 eV [38]. Moreover, the concentrations of C–O and O=C–O listed in Figure 7a were 8.85 at.% and 3.17 at.%, respectively. As shown in Figure 7b, after Friedel–Crafts alkylation, the concentration of C-O on F-PPTA surface significantly increased to 24.92 at.%. Combined with the FTIR test results mentioned above, it could be confirmed that the epoxy groups were formed on the surface of fibers. Compared with Figure 7b, the concentration of O=C–O in Figure 7c increased from 3.02 at.% to 5.31 at.%, which further indicated that COOH-MWCNTs were chemically deposited on the F-PPTA surface. Namely, there were ester groups formed through the reaction between epoxy groups and carboxyl groups as shown in the mechanism of Figure 2. Besides, the concentration of C–O was almost unchanged, which illustrated the formation of hydroxyl groups.

### 3.4. Surface Energy Analysis of Fibers

In order to study the wettability of PPTA fibers before and after deposition of MWCNTs, the contact angles were measured by drop length–height method to calculate the surface energy. The test liquids were water as the polar solvent and hexane as the nonpolar solvent, which had a large difference in surface energy components.

The relationship between surface energy (*γ*) and contact angle (*θ*) could be obtained by Young–Dupre equation [39], as shown in Equation (1).

(1) $$\gamma_{LS}=\gamma_{S}-\gamma_{L}\cos\theta$$

where, $\gamma_{L}$, $\gamma_{S}$ and $\gamma_{SL}$ are the surface energy of liquid, solid and liquid–solid interface, respectively.

According to the surface energy component method of Owens and Wendt [40], the liquid–solid interfacial energy could be obtained by the Equations (2) and (3).

(2) $$\gamma_{LS}=\gamma_{S}+\gamma_{L}-2\sqrt{\gamma_{S}^{p}\gamma_{L}^{p}}-2\sqrt{\gamma_{S}^{d}\gamma_{L}^{d}}$$

(3) $$\gamma_{S}=\gamma_{S}^{p}+\gamma_{S}^{d},\;\gamma_{L}=\gamma_{L}^{p}+\gamma_{L}^{d}$$

In Equations (2) and (3), $\gamma_{S}^{p}$ and $\gamma_{L}^{p}$ are polar components of surface energy of solid and liquid, respectively. $\gamma_{S}^{d}$ and $\gamma_{L}^{d}$ are nonpolar components of surface energy of solid and liquid, respectively.

Combined with the Equations (1) and (2), the Equation (4) could be obtained as follows.

(4) $$\gamma_{L}\left(\cos\theta +1\right)=2\sqrt{\gamma_{S}^{p}\gamma_{L}^{p}}+2\sqrt{\gamma_{S}^{d}\gamma_{L}^{d}}$$

Based on the tests of contact angles, the surface energy of PPTA fibers ($\gamma_{S}$) could be calculated by Equations (3) and (4).

**Figure 8:**
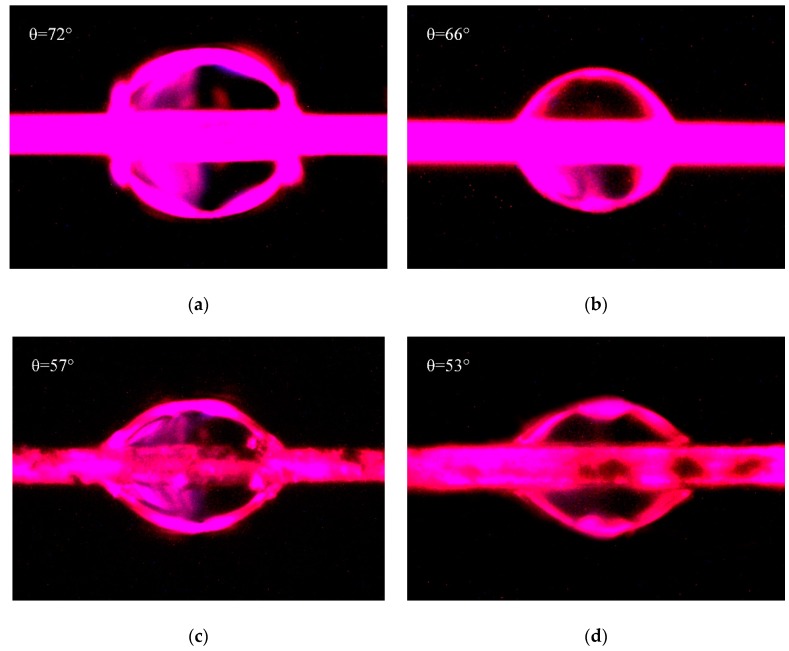
Contact angles of PPTA fibers: (**a**) A-PPTA with water; (**b**) A-PPTA with hexane; (**c**) MWCNTs-PPTA with water; (**d**) MWCNTs-PPTA with hexane.

The micrographs of contact angles measured by drop length-height method were shown in Figure 8. Figure 8a,c were both measured with water, and Figure 8b,d were both measured with hexane. Due to the penetration of liquid into fibers, the contact angles would decrease with the increase of soaking time. Therefore, in this test, the micrographs of these contact angles were photographed at the similar time in order to decrease the measuring error. The contact angles as shown in Figure 8 were fitted and measured by Young–Laplace software. Combined with $\gamma_{L}^{p}$ and $\gamma_{L}^{d}$ of water are 51 mJ/m^2^ and 21.8 mJ/m^2^, and those of hexane are 0 mJ/m^2^ and 18.4 mJ/m^2^ [41], the surface energies of PPTA fibers were calculated by Equations (3) and (4), and the results were summarized in Table 2.

Table 2 shows that after the deposition of MWCNTs, the contact angle of PPTA fibers with water decreased from 72° to 57°, and that of PPTA fibers with hexane decreased from 66° to 53°. Meanwhile, the surface energy of fibers increased remarkably by 41.9% (from 31 mJ/m^2^ to 44 mJ/m^2^). It could also be obtained from Table 2 that the polar component of surface energy ($\gamma_{S}^{p}$) of PPTA fibers was the main part for the increase of surface energy. This was because the ports of MWCNTs had a large number of polar groups, which could effectively reinforce the dipole–dipole interaction with polar solvent [24]. In addition, after chemical deposition of MWCNTs, the newly formed hydroxyl groups and the residual carboxyl groups could also enhance the hydrogen bonding with polar solvent [42]. Meanwhile, the increased surface roughness of MWCNTs-PPTA could slightly improve the Van Der Waals Force between the fibers and the non-polar solvent, which resulted in enhancing the non-polar component of surface energy ($\gamma_{S}^{d}$). It could be indicated from the significant increase of surface energy that the deposited fibers had a better wettability and would show outstanding adhesive performance with the rubber matrix.

### 3.5. Mechanical and Adhesive Properties

As the reinforced layer of rubber matrix, the strength of PPTA fibers played a crucial role in the integral strength of composites. The tensile strength of fibers might decrease with the increase of gauge length due to the distribution of defects [43]. Thus, the same gauge length of 20 mm was selected to reduce the measuring error. The average tensile strength and elongation rate of PPTA fibers as shown in Table 3 were measured by electronic tensile tester of single-filament. The tensile strength ($\sigma_{f}$) could be calculated by the following Equation (5) [44].

(5) σf=Fmaxπd24

where, $F_{max}$ and *d* are the maximum fracture load and diameter of fiber, respectively.

The elongation at break (ε) could be calculated by the following Equation (6).

(6) $$\varepsilon =\frac{\Delta l}{l}$$

where, Δl and *l* are the elongation length at break and the gauge length, respectively.

It could be found from Table 3 that the tensile strength and elongation rate had light change before and after deposition, which suggested that the deposition method of MWCNTs used in this study might reduce the mechanical properties of PPTA fibers slightly. The reason for this might be illustrated by the damage of molecular chain by alkali treatment during the modification. Meanwhile, under the ultrasonic, PPTA fibers would be swelled in NMP and small amounts of MWCNTs could be incorporated into fibers to improve the mechanical properties [23], namely weaken the adverse effect of alkali treatment. 

In order to study the adhesive properties of PPTA fibers with rubber matrix, the pull-out force and peeling strength were tested and the results were also summarized in Table 3. After the chemical deposition of MWCNTs, the pull-out force increased by 46.3% (from 28.3 N to 41.4 N), and the peeling strength increased by 56.5% (from 2.3 N/m to 3.6 N/m), which indicated that the adhesive properties of PPTA fibers with rubber had been greatly enhanced.

**Figure 9:**
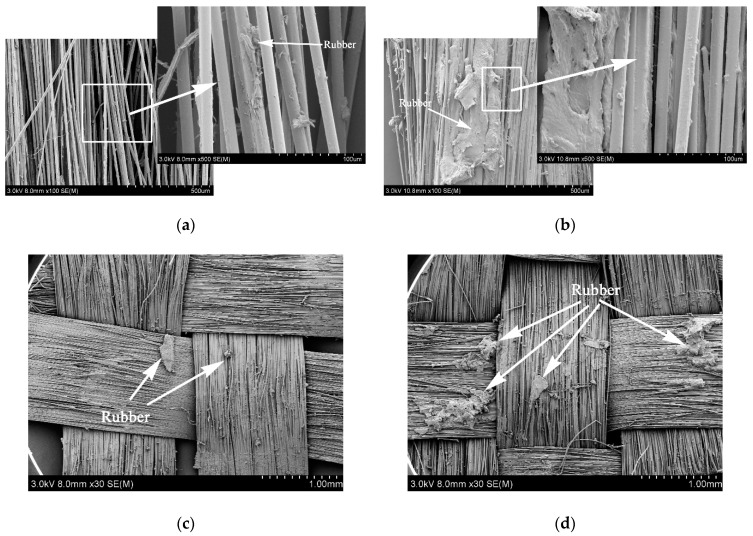
Surface morphologies of the PPTA fibers after adhesive properties tests: (**a**) A-PPTA after pull-out force test; (**b**) MWCNTs-PPTA after pull-out force test; (**c**) A-PPTA after peeling strength test; (**d**) MWCNTs-PPTA after peeling strength test.

Figure 9 shows the surface morphology of PPTA fibers after the tests of pull-out force and peeling strength. From Figure 9a, it could be observed that there was little rubber remaining on the A-PPTA fibers surface, namely, the fracture point was located at the interface between the fibers and the rubber. Besides, the fibers were independent of each other, and the interface was clean with poor adhesive property. In comparison with this, the surface of MWCNTs-PPTA fibers after pull-out force test had more obvious residual rubber as shown in Figure 9b. The fracture point was located at the rubber matrix. In addition, there was more rubber remaining between the fibers and the adhesive property was outstanding. Similarly, it could be found from Figure 9c,d that after peeling strength test, the residual rubber of A-PPTA fiber cloth was in granular shape while that of MWCNTs-PPTA fiber cloth was in block shape, which proved that the PPTA fibers after deposition had a better adhesive property with rubber. 

**Figure 10:**
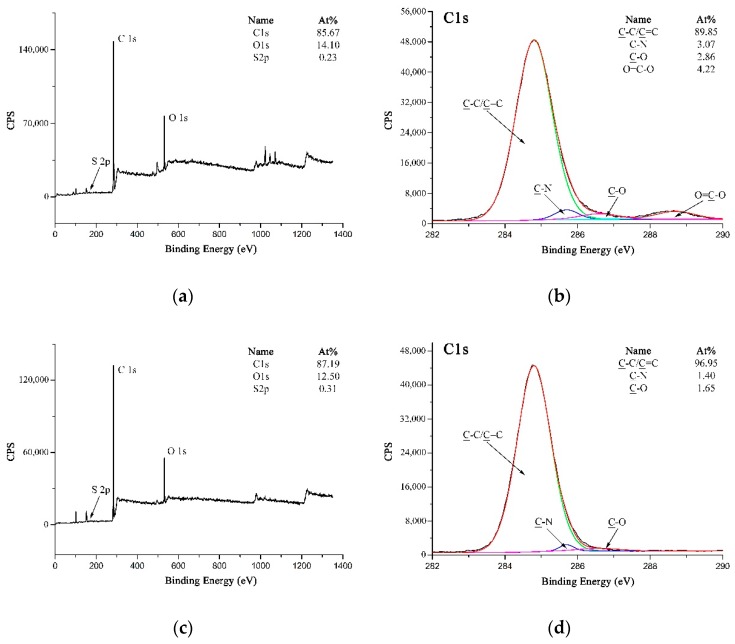
XPS spectra: (**a**) Wide-scan spectrum of the rubber matrix; (**b**) High-resolution C 1s spectrum of the rubber matrix; (**c**) Wide-scan spectrum of MWCNTs-PPTA after peeling strength test; (**d**) High-resolution C 1s spectrum of MWCNTs-PPTA after peeling strength test.

Theoretically, due to the homolysis reaction during the vulcanization process, sulfur was turned into sulfur radicals which would react with the double bonds of rubber to generate the mercaptan groups [45]. Similar with the hydroxyl groups, the mercaptan groups could also react with the carboxyl groups to form the thioester groups [46]. However, it could be obtained from the wide-scan spectra of the rubber matrix and MWCNTs-PPTA after peeling strength test (as shown in Figure 10a,c) that the sulfur concentration was too low to form the covalent bonds on the surface of fibers. Figure 10b could be fitted with three peaks, which were attributed to C–C/C=C at 284.8 eV, C–N at 285.7 eV and C–O at 286.6 eV. Compared with this, there was a new peak at 288.7 eV in the high-resolution C 1s spectrum of MWCNTs-PPTA after peeling strength test (as shown in Figure 10d), which was attributed to the residual O=C–O on the surface of MWCNTs-PPTA fibers. Thus, there was no new group (such as the thioester group) formed in the adhesion process between the fibers and the rubber. Therefore, the reasons for the enhancement of adhesive properties might be illustrated by the following two non-covalent aspects. Firstly, the increase of surface energy could promote the affinity of PPTA fibers to the rubber matrix. Secondly, the chemical deposited MWCNTs could form network structures on the surface of PPTA fibers, which would increase the contact area and generate mechanical interlocking with the rubber [47].

## 4. Conclusions

MWCNTs were uniformly deposited onto the surface of PPTA fibers with the formation of covalent bonds in an attempt to improve the adhesive properties between the fibers and the rubber matrix. The chemical method used in this study was novel and efficient. The surface energy of the fibers increased by 41.9%, which significantly improved the wettability. Depending on the study on the mechanical properties of PPTA fibers, this deposition method just reduced the single-filament tensile strength slightly. Furthermore, the pull-out force and peeling strength of PPTA fibers after deposition with the rubber matrix increased by 46.3% and 56.5%, respectively, which meant that the interfacial adhesion between the fibers and the rubber was improved remarkably. It could be predicted that this novel chemical deposition method would apply to more kinds of matrix and expand the application range of PPTA fibers.

## Figures and Tables

**Table 1:** Rubber formulation.

Materials	Parts per Hundreds of Rubber
Styrene-butadiene rubber	60
Natural rubber	40
Antioxidant (4010NA)	1.5
Carbon black	20
White carbon black	15
Zinc oxide	5
Stearic acid	2.5
Aromatic oil	10
Coumarone indene resin	10
Rubber adhesive (RA)	1
Rubber adhesive (RS)	1
Accelerant (CZ)	5
Sulphur	1
Total	172

**Table 2:** Surface energies of PPTA fibers.

Fibers	Contact Angle (°)		Surface Energy (mJ/m^2^)		
	Water	Hexane	$\gamma_{S}^{p}$	$\gamma_{S}^{d}$	$\gamma_{S}$
A-PPTA	72 ± 2	66 ± 1	22	9	31
MWCNTs-PPTA	57 ± 1	53 ± 1	32	12	44

**Table 3:** The single-filament mechanical properties of PPTA fibers and adhesive strength with rubber.

Fibers	Tensile Strength (GPa)	Elongation at Break (%)	Pull-Out Force (N)	Peeling Strength (N/mm)
PPTA	3.9 ± 0.07	3.5 ± 0.2	28.3 ± 1.8	2.3 ± 0.1
MWCNTs-PPTA	3.7 ± 0.07	3.3 ± 0.2	41.4 ± 1.7	3.6 ± 0.1
